# *MPRIP::PDGFRB* fusion identified in a male patient with a myeloid/lymphoid neoplasm with eosinophilia

**DOI:** 10.1007/s00277-026-07035-8

**Published:** 2026-04-27

**Authors:** Min Gao, Kimo Bachiashvili, Omer Jamy, Shuko Harada, Alexander Craig Mackinnon, Aishwarya Ravindran, Yunjia Chen, Andrew J. Carroll, Fady M. Mikhail

**Affiliations:** 1 Department of Genetics , University of Alabama at Birmingham, Birmingham, AL USA; 2 Department of Medicine (Hematology/Oncology) , University of Alabama at Birmingham, Birmingham, AL USA; 3 Department of Pathology , University of Alabama at Birmingham, Birmingham, AL USA

**Keywords:** Myeloid/lymphoid neoplasms with eosinophilia (MLN-eo), *PDGFRB* rearrangement, *MPRIP*:*PDGFRB* fusion, Imatinib

## Abstract

Myeloid/lymphoid neoplasms with eosinophilia (MLN-eo) associated with *PDGFRB* rearrangements represent a rare but clinically significant category of hematologic malignancies, characterized by clonal eosinophilia and exceptional sensitivity to tyrosine kinase inhibitors. Among these, the *MPRIP*::*PDGFRB* fusion is exceptionally uncommon, with only three patients previously reported. Here, we report the first United States patient with *MPRIP*::*PDGFRB* fusion in a 34-year-old man presenting with leukocytosis, marked eosinophilia, anemia, thrombocytopenia, mucocutaneous lesions, hepatosplenomegaly, chronic gastrointestinal symptoms, and tree-in-bud pulmonary nodularity. Bone marrow evaluation revealed a markedly hypercellular marrow with increased eosinophils, grade 1 reticulin fibrosis, and dysmegakaryopoiesis. Fluorescence in situ hybridization (FISH) demonstrated a *PDGFRB* rearrangement in 76% of cells, and chromosome analysis revealed a balanced translocation t(5;17)(q32;p11.2). Next-generation sequencing RNA-based myeloid fusion panel analysis identified an *MPRIP*::*PDGFRB* fusion formed by an in-frame junction of *MPRIP* exon 20 and *PDGFRB* exon 12. The patient was initiated on imatinib therapy and achieved rapid remission. To contextualize this patient, we compared the clinical and molecular features of all previously published patients with *MPRIP*::*PDGFRB* fusion, noting shared findings of marked eosinophilia and excellent imatinib sensitivity, but notable heterogeneity in symptom burden, degree of marrow fibrosis, and associated immune-related manifestations. Additionally, we provide an overview of 45 reported *PDGFRB* fusion partner genes, summarizing their cytogenetic characteristics, and associated diseases. This case report expands the clinical spectrum of *MPRIP*::*PDGFRB* positive MLN-eo and underscores the essential role of cytogenetic and molecular testing in the diagnostic evaluation of eosinophilia, given the significant therapeutic implications of identifying *PDGFRB* fusions.

**Supplementary Information:**

The online version contains supplementary material available at 10.1007/s00277-026-07035-8.

## Introduction

 Eosinophilic disorders encompass a broad spectrum ranging from reactive responses to clonal hematopoietic neoplasms. In recognition of recurrent, molecularly defined cases, the WHO classification formally established the “myeloid/lymphoid neoplasms with eosinophilia (MLN-eo) and rearrangement of *PDGFRA*, *PDGFRB*, *FGFR1* or *PCM1*::*JAK2 *fusion” entity as a discrete disease category [1]. These genetically defined neoplasms are driven by fusion-activated tyrosine kinases that confer a clonal proliferation advantage to the eosinophil-lineage or other hematopoietic progenitors [2, 3]. Clinically, MLN-eo may present with variable degrees of eosinophilia, bone marrow hypercellularity, and multi-organ involvement, including dermatologic, pulmonary, gastrointestinal, hepatic, or splenic manifestations secondary to eosinophilic infiltration or mediator-driven tissue injury [2, 4]. Importantly, these disorders exhibit dramatic responses to tyrosine kinase inhibitors (TKIs), underscoring the necessity of comprehensive cytogenetic and molecular testing in patients with unexplained eosinophilia.

Among the MLN-eo subtypes, *PDGFRB*-rearranged neoplasms are rare but clinically significant due to their exquisite sensitivity to TKIs such as imatinib. More than 45 *PDGFRB* fusion partners have been described to date, most arising from balanced translocations involving chromosome band 5q32, which juxtapose the *PDGFRB* kinase domain with diverse partner genes. Reported partners include *ETV6*, *TRIP11*, *CCDC6*, *RABEP1*, *PDE4DIP*, *SPECC1*, *NDE1*, *MYO18A*, and others. These genes typically provide oligomerization domains that promote constitutive *PDGFRB* kinase activation, driving clonal myeloproliferation [5–17]. *MPRIP* (myosin phosphatase Rho-interacting protein) regulates cytoskeletal dynamics by targeting myosin phosphatase to control myosin light chain phosphorylation [18]. Recurrent *MPRIP* fusion events have been reported across multiple malignancies, including *MPRIP*::*NTRK1*, *MPRIP*::*ALK* and *MPRIP*::*ROS1* in non-small cell lung cancer [19]. The *MPRIP*::*PDGFRB* fusion is exceptionally uncommon, with only three cases previously reported worldwide [5, 20, 21]. This fusion typically results from the translocation t(5;17)(q32;p11.2), which produces a chimeric fusion protein that constitutively activates *PDGFRB* signaling. Reported patients consistently exhibit marked eosinophilia, bone marrow eosinophilic hyperplasia, splenomegaly, and robust responses to low-dose imatinib.

In this report, to our knowledge, we describe the first documented patient of *MPRIP::PDGFRB* fusion positive MPN-eo in the United States, affecting a 34-year-old man with multisystem involvement. The other three reported patients were from Germany, Thailand, and Belgium [5, 20, 21]. We compare his clinical, hematologic, and molecular findings with previously published cases (Table 1), and provide an updated overview of 45 known *PDGFRB* fusion partner genes (Table 2), emphasizing their cytogenetic features and disease associations. This case contributes to the expanding clinical spectrum of *PDGFRB*-rearranged neoplasms and underscores the critical role of cytogenetic and molecular testing in the evaluation of eosinophilia to enable timely diagnosis and initiation of effective targeted therapy.

**Table 1:** Clinical characteristics of the current patient and previously published patients with *MPRIP*::*PDGFRB* fusion

Sex	Current patient	Ukkahad et al., 2025	Beel et al., 2021	Naumann et al., 2015
	Male	Male	Male	Male
Age (at testing)	34 years	32 years	37 years	80 years
Hematologic	Leukocytosis with neutrophilia and eosinophilia; anemia; thrombocytopenia	Hypereosinophilia; mild anemia	Leukocytosis with marked eosinophilia; mild anemia	Leukocytosis with eosinophilia; normal platelets
Peripheral Blood	WBC 4.94 × 10¹⁰/L; neutrophils 75% (3.705 × 10¹⁰/L); lymphocytes 1% (4.94 × 10⁸/L); monocytes 4% (1.976 × 10⁹/L); eosinophils 13% (6.422 × 10⁹/L); basophils 1% (4.94 × 10⁸/L); myelocytes 6% (2.964 × 10⁹/L); Hb 105 g/L; Plt 1.095 × 10¹¹/L	WBC 3.33 × 10¹⁰/L; neutrophils 40% (1.332 × 10¹⁰/L); lymphocytes 17% (5.661 × 10⁹/L); monocytes 10% (3.33 × 10⁹/L); eosinophils 20% (6.66 × 10⁹/L); basophils 2% (6.66 × 10⁸/L); promyelocytes 3% (9.99 × 10⁸/L); myelocytes 3% (9.99 × 10⁸/L); metamyelocytes 5% (1.665 × 10⁹/L); Hb 103 g/L; Plt 8.0 × 10¹⁰/L	WBC 1.29 × 10¹⁰/L; eosinophils 39.6% (5.11 × 10⁹/L); myelocytes 3.6% (4.64 × 10⁸/L); metamyelocytes 1.8% (2.32 × 10⁸/L); Hb 112 g/L; Vitamin B12 > 2000 ng/L; IgE elevated	WBC 1.30 × 10¹⁰/L; neutrophils 22% (2.86 × 10⁹/L); monocytes 5% (6.50 × 10⁸/L); eosinophils 29% (3.77 × 10⁹/L); myelocytes 5% (6.50 × 10⁸/L); Hb 140 g/L; Plt normal
Bone Marrow	Markedly hypercellular (> 95%); eosinophils 30%; Grade 1 reticulin fibrosis; dysmegakaryopoiesis; blasts < 5%	Hypercellular marrow (95%); granulocytic hyperplasia; eosinophils 80%; decreased megakaryocytes; Grade 2 reticulin fibrosis; no increased blasts	Hypercellular marrow; blasts < 5%	Hypercellular; no eosinophils reported; micromegakaryocytes; blasts < 5%
*PDGFRB* Rearrangement (%)	79%	Not specified	73%	70%
Dermatologic	Folliculitis/pustular rash involving scalp, neck, chest, and back	Intermittent pruritus without rash	Pollen allergy, no skin disease reported	Not reported
Pulmonary	Tree-in-bud nodular opacities in both lungs	Not reported	Not reported	Not reported
Gastrointestinal	Soft, non-tender abdomen; normal bowel sounds; 8–10 months of chronic loose stools	No GI involvement aside from weight loss; normal liver imaging	Not reported	Not reported
Hepatosplenic	Hepatosplenomegaly	Splenomegaly	Not reported	Splenomegaly
Mucosal / Immune	Oropharyngeal candidiasis; tongue ulcer	Not reported	Not reported	Not reported
Constitutional Symptoms	No B symptoms	Fever, night sweats, 6‑kg weight loss	None reported	None
Treatment	Imatinib (*PDGFRB*-positive MPN-eo); Doxycycline (folliculitis); Allopurinol (tumor-lysis prophylaxis); Nystatin (oral candidiasis); Acyclovir (antiviral prophylaxis)	Imatinib	Imatinib	Imatinib
Response	Rapid remission; reassessment and dose reduction	Remission at 1y	Remission at 1y	Remission at 6 m

Note: *WBC* White blood cell count, *Hb* Hemoglobin, *Plt* Platelet count, *IgE* Immunoglobulin E, *MPN-eo* Myeloproliferative neoplasm with eosinophilia, *B12 *Vitamin B12,  *MF *Myelofibrosis, *B *symptoms, Fever, night sweats, weight loss,  *y * year, *m* month

**Table 2:** Overview of 45 *PDGFRB* fusion partner genes

No.	Partner (gene)	Chromosome aberration	Disease	Reference	
1	*ETV6* (*TEL*)	t(5;12)(q33;p13)	CMML / MPN-eo	Golub et al., 1994	
2	*TRIP11* (*CEV14*)	t(5;14)(q33;q32)	AML	Abe et al., 1997	
3	*HIP1*	t(5;7)(q33;q11.2)	CMML	Ross et al., 1998	
4	*CCDC6* (*H4*)	t(5;10)(q33;q21)	MPN	Kulkarni et al., 2000	
5	*RABEP1*	t(5;17)(q33;p13)	CMML	Magnusson et al., 2001	
6	*PDE4DIP*	t(1;5)(q23;q33)	MDS/MPN	Wilkinson et al., 2003	
7	*NIN*	t(5;14)(q33;q24)	aCML	Vizmanos et al., 2004	
8	*SPECC1* (*HCMOGT1*)	t(5;17)(q33;p11.2)	JMML	Morerio et al., 2004	
9	*TP53BP1*	t(5;15)(q33;q22)	aCML	Grand et al., 2004	
10	*CCDC88C* (*KIAA1509*)	t(5;14)(q33;q32)	MPN	Levine et al., 2005	
11	*TPM3*	t(1;5)(q21;q33)	CEL	Rosati et al., 2006	
12	*NDE1*	t(5;16)(q33;p13)	CMML	La Starza et al., 2007	
13	*CAPRIN1* (*GPIAP1*)	der(1)t(1;5), der(5)t(1;5), der(11)ins(11;5)	MPN	Walz et al., 2007	
14	*GIT2*	t(5;12)(q31-33;q24)	MPN	Walz et al., 2007	
15	*PRKG2*	t(4;5;5)(q23;q31;q33)	MPN	Walz et al., 2007	
16	*SPTBN1*	t(2;5)(p21;q33)	aMPN	Gallagher et al., 2008	
17	*ERC1*	t(5;12)(q33;p13.3)	AML	Gorello et al., 2008	
18	*WDR48*	t(1;3;5)(p36;p21;q33)	aMPN	Hidalgo-Curtis et al., 2009	
19	*GOLGA4*	t(3;5)(p21;q31)	aCML	Hidalgo-Curtis et al., 2009	
20	*BIN2*	t(5;12)(q33;q13)	aMPN	Hidalgo-Curtis et al., 2009	
21	*MYO18A*	t(5;17)(q33-34;q11.2)	CEL	Walz et al., 2009	
22	*SART3*	t(5;12)(q31-32;q23-24)	CEL	Erben et al., 2010	
23	*KANK1*	t(5;9)(q31-32;p22-24.3)	MPN	Medves et al., 2010	
24	*CEP85L*	t(5;6)(q33-34;q23)	T-ALL/MPN	Chmielecki et al., 2012	
25	*EBF1*	*EBF1* ex1-14 (15); *PDGFRB* ex11-23	B-ALL	Roberts et al., 2012	
26	*TNIP1*	none	CEL	Moshir et al., 2012	
27	*NDEL1*	t(5;17)(q33;p13.1)	JMML	Byrgazov et al., 2014	
28	*DTD1*	t(5;20)(q33;p12)	CEL	Gosenca et al., 2014	
29	*ATF7IP*	t(5;12)(q33;p13)	B-ALL	Kobayashi et al., 2014	
30	*CPSF6*	t(5;12)(q33;q15)	MPN-eo	Naumann et al., 2015	
31	*GOLGB1*	t(3;5)(q13;q33)	MPN-eo	Naumann et al., 2015	
32	*NUMA1*	t(5;11)(q32;q13.4)	MPN-eo	Zou et al., 2017	
33	*AGGF1*	*AGGF1* ex1-10; *PDGFRB* ex11-23	ALL	Zabriskie et al., 2018	
34	*GTF2I*	t(5;12)(q33;p13)	Ph-like ALL	Panagopoulos et al., 2019	
35	*CSNK2A1*	*CSNK2A1* ex1-4; *PDGFRB* ex12-23	MPN-eo	Xu et al., 2020	
36	*WNK1*	t(5;12)(q33;p13)	MLN-eo	Wang et al., 2022	
37	*PCM1*	t(5;8)(q32;p22)	MDS/MPN	Wang et al., 2022	
38	*NRIP1*	*NRIP1* ex1-3; *PDGFRB* ex12-23	Ph-like ALL	Miyazaki et al., 2023	
39	*CD74*	*CD74* intr1; *PDGFRB* ex11-23	Ph-like ALL	Sadras et al., 2023	
40	*TPR*	*TPR* ex1-44; *PDGFRB* ex12-23	Ph-like ALL	Zhang et al., 2023	
41	*TERF2*	*TERF2* ex1-8; *PDGFRB* ex9-23	Ph-like ALL	Xu et al., 2024	
42	*MYH9*	*MYH9* ex1-25; *PDGFRB* ex10-23	T-LBL	De Coninck et al., 2024	
43	*UBE2I*	t(5;6)(q32;p13.3)	IMF	Boccia et al., 2025	
44	*FN1*	t(2;5)(q35;q32)	IMF	Boccia et al., 2025	
45	*MPRIP*	t(5;17)(q33;p11)	MPN-eo		Naumann et al., 2015; Beel et al., 2021; Ukkahad et al., 2025; Current patient

Note: *CMML* Chronic myelomonocytic leukemia, *MPN-eo *Myeloproliferative neoplasm with eosinophilia, *AML* Acute myeloid leukemia, *MPN * Myeloproliferative neoplasm,  *MDS/MPN * Myelodysplastic/myeloproliferative neoplasm, *aCML *Atypical chronic myeloid leukemia,  *JMML *Juvenile myelomonocytic leukemia,  *CEL *Chronic eosinophilic leukemia, *aMPN *Atypical myeloproliferative neoplasm, *T-ALL/MPN *Mixed T-acute lymphoblastic leukemia/myeloproliferative neoplasm, * B-ALL*: B-acute lymphoblastic leukemia, *ALL *Acute lymphoblastic leukemia,  *Ph-like ALL * Philadelphia chromosome–like acute lymphoblastic leukemia,  *MLN-eo * Myeloid/lymphoid neoplasm with eosinophilia,  *T-LBL *T-lymphoblastic lymphoma, *IMF* Infantile myofibromatosis,  *ex* exon, *intr *intron

## Materials and methods

### Flow cytometry and bone marrow analysis

Peripheral blood and bone marrow aspirate specimens from our patient underwent comprehensive diagnostic evaluation at the University of Alabama at Birmingham (UAB). Peripheral blood analysis included automated cell counts with smear examination to assess leukocyte differentials, eosinophil burden, circulating blasts, and morphologic abnormalities. Bone marrow aspirate specimen underwent multiparameter flow cytometry using a standardized antibody panel to evaluate hematopoietic lineages. Bone marrow examination also included morphologic assessment of aspirate smears and core biopsy sections to determine overall cellularity, lineage distribution, eosinophil proportion, dysplasia, blast percentage, and reticulin fibrosis. All findings were interpreted in conjunction with clinical and laboratory data to characterize marrow involvement.

### G-banded chromosome and fluorescence in situ hybridization (FISH) analyses

Bone marrow specimen from our patient underwent comprehensive cytogenomic evaluation, including G-banded chromosome analysis and fluorescence in situ hybridization (FISH), performed at the UAB Cytogenetics Laboratory. Conventional karyotyping was carried out using standard G-banding protocols. Interphase FISH studies were performed according to the manufacturer’s instructions and established laboratory procedures, as previously described [22]. The eosinophilia FISH panel included the 4q12 tri-color probe (Abbott Molecular) for detecting the interstitial deletion generating the *FIP1L1*::*PDGFRA* fusion, along with dual-color break-apart probes (BAP) (Cytocell) targeting *PDGFRB* (5q32), *FGFR1* (8p11.2), *JAK2* (9p24.1), and *ETV6* (12p13.2). All cytogenomic findings were interpreted and reported in accordance with the International System for Human Cytogenomic Nomenclature (ISCN) 2024.

### Targeted next-generation sequencing (NGS) panel analysis

Next-generation sequencing (NGS) was performed on bone marrow aspirate from our patient using both Oncomine myeloid RNA assay GX v2 and Oncomine myeloid DNA assay GX v2 (Thermo Fisher). The myeloid targeted RNA panel interrogated a broad range of recurrent fusion driver genes, including *ABL1*, *ABL2*, *BCL2*,* BRAF*,* CCND1*,* CREBBP*,* EGFR*,* ETV6*,* FGFR1*,* FGFR2*,* FUS*,* HMGA2*,* JAK2*,* KAT6A (MOZ)*,* KAT6B*,* KMT2A/KMT2A-PTDs*,* MECOM*,* MET*,* MLLT10*,* MKL1*,* MYBL1*,* MYH11*,* NTRK2*,* NTRK3*,* NUP214*,* NUP98*,* PAX5*,* PDGFRA*,* PDGFRB*,* RARA*,* RUNX1*,* TCF3*,* TFE3*, and *ZNF384*. Total RNA was extracted from bone marrow cells and analyzed by targeted NGS. Detected fusion transcripts were annotated using validated databases and interpreted in the context of the patient’s clinical and pathologic findings. In parallel, genomic DNA underwent testing with the Oncomine Myeloid Comprehensive DNA Panel, which assesses hotspot mutations in 28 genes (*ABL1*,* ANKRD26*,* BRAF*,* CBL*,* CSF3R*,* DDX41*,* DNMT3A*,* FLT3*,* GATA2*,* HRAS*,* IDH1*,* IDH2*,* JAK2*,* KIT*,* KRAS*,* WT1*,* MPL*,* MYD88*,* NPM1*,* NRAS*,* PPM1D*,* PTPN11*,* SETBP1*,* SF3B1*,* SMC1A*,* SMC3*,* SRSF2*, and *U2AF1*), as well as full coding sequences of 17 additional genes (*ASXL1*,* BCOR*,* CALR*,* CEBPA*,* ETV6*,* EZH2*,* IKZF1*,* NF1*,* PHF6*,* PRPF8*,* RB1*,* RUNX1*,* SH2B3*,* STAG2*,* TET2*,* TP53*, and *ZRSR2*). Library preparation, sequencing, variant calling, and annotation followed standardized clinical protocols, and all results were interpreted by board-certified molecular pathologists according to AMP/ASCO/CAP guidelines [23].

### Case presentations

A 34-year-old man with no significant past medical history presented with several months of progressive fatigue, a pruritic pustular rash involving the scalp, neck, chest, and back, and chronic loose stools lasting 8–10 months. Physical examination revealed oral thrush, aphthous ulceration of the tongue, and hepatosplenomegaly. His gastrointestinal examination showed a soft, non-tender abdomen with normal bowel sounds. Constitutional symptoms such as fever, night sweats, or weight loss were not reported. Imaging of the neck, chest, abdomen and pelvis with CT scans demonstrated cervical adenopathy, hepatosplenomegaly, and bilateral tree-in-bud nodular opacities despite the absence of respiratory symptoms.

Initial laboratory evaluation demonstrated leukocytosis with neutrophilia, eosinophilia, anemia, and thrombocytopenia. Hematologic evaluation revealed marked leukocytosis with a white blood cell count of 4.94 × 10¹⁰/L, consisting of 75% (3.705 × 10¹⁰/L) neutrophils, 13% (6.422 × 10⁹/L) eosinophils, 1% (4.94 × 10⁸/L) basophils, 1% (4.94 × 10⁸/L) lymphocytes, 4% (1.976 × 10⁹/L) monocytes, and 6% (2.964 × 10⁹/L) myelocytes. Hemoglobin was 105 g/L, and platelet count was 1.095 × 10¹¹/L, indicating concurrent anemia and thrombocytopenia. Work-up for infectious, allergic and rheumatological etiologies of eosinophilia was negative. Bone marrow biopsy demonstrated a markedly hypercellular marrow (> 95% cellularity) with 30% eosinophils, grade 1 reticulin fibrosis, and dysmegakaryopoiesis, with fewer than 5% blasts and no circulating blasts (Table S1).

**Fig. 1:**
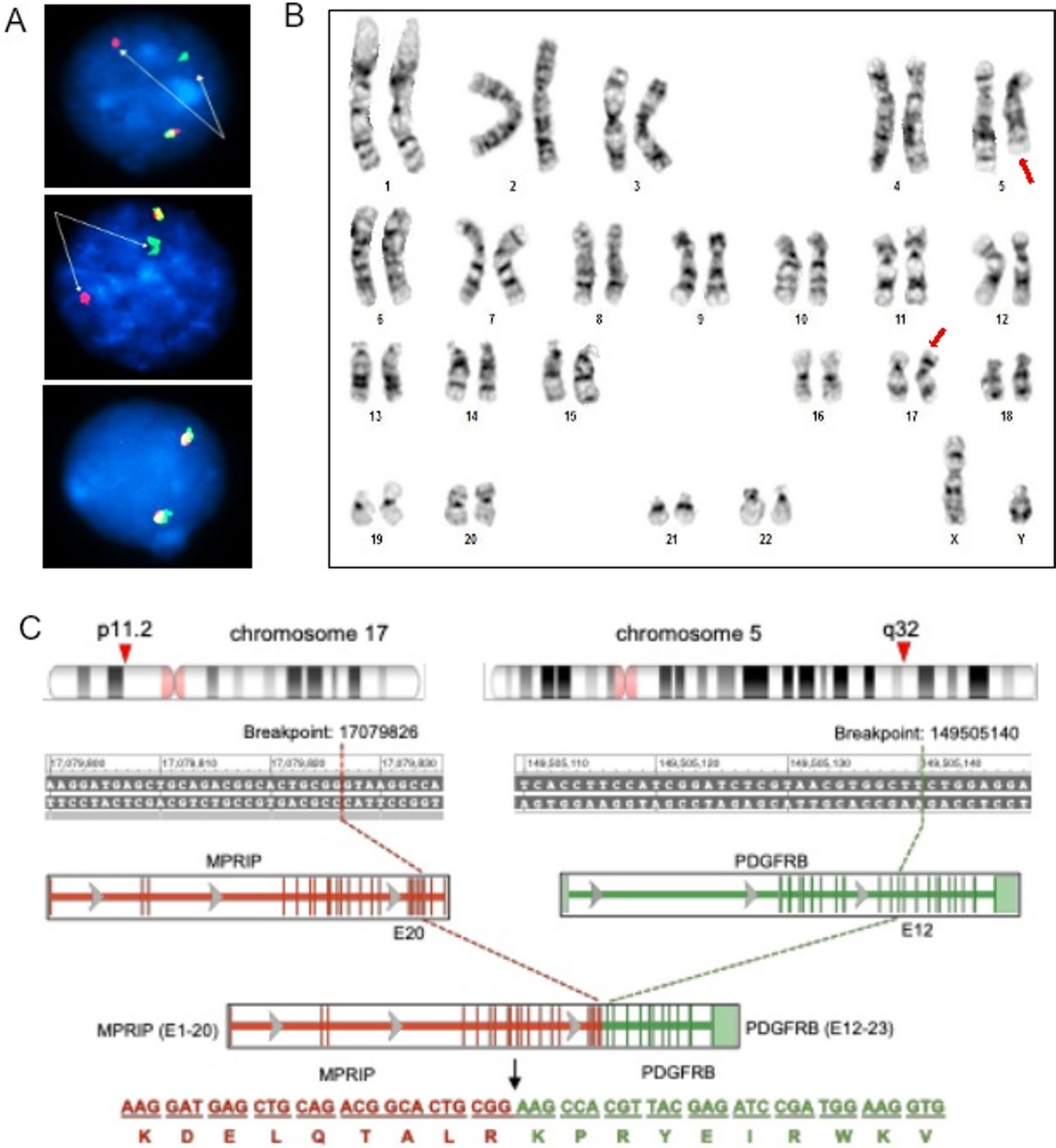
(**A**) Interphase FISH analysis of unstimulated bone marrow (BM) cells of the patient using the *PDGFRB* BAP, the upper and middle panels demonstrate *PDGFRB* rearrangement, indicated by separation of the red and green signals (white arrows). The lower panel shows a normal (non-rearranged) signal pattern. (**B**) G-banded chromosome analysis of unstimulated BM cells of the patient, demonstrating a balanced translocation involving chromosome 5 and 17 with breakpoints at bands 5q32 and 17p11.2 [t(5;17)(q32;p11.2)]. Red arrows indicate the derivative chromosomes involved in the translocation. (**C**) Schematic representation of *MPRIP*::*PDGFRB* fusion. Breakpoints are mapped to chromosome 17p11.2 (*MPRIP* exon 20) and chromosome 5q32 (*PDGFRB* exon 12), resulting in an in-frame fusion of *MPRIP* (exons 1–20) to *PDGFRB* (exons 12–23). The predicted chimeric fusion protein sequence at the junction is shown below

Interphase FISH performed on the patient’s bone marrow using the *PDGFRB* BAP confirmed a *PDGFRB* rearrangement at 5q32 in 79% of analyzed cells (Fig. 1A). The abnormal pattern demonstrated clear separation of the red 5′ and green 3′ signals, consistent with a classic *PDGFRB* gene rearrangement, while a normal fusion signal pattern was observed in the remaining cells. Parallel FISH assays targeting *PDGFRA*, *FGFR1*, *JAK2*, and *ETV6* revealed no evidence of rearrangements in these cells (Table S1). Subsequent G-banded karyotyping revealed a clonal balanced translocation between chromosomes 5 and 17 [t(5;17)(q32;p11.2)], and the karyotype was reported as 46,XY,t(5;17)(q32;p11.2)[18]/46,XY[2] (Fig. 1B). Together, these cytogenomic findings established the presence of a *PDGFRB* rearrangement as a result of the t(5;17)(q32;p11.2).

RNA-based NGS using the Myeloid Fusion Panel performed on the patient’s bone marrow identified an *MPRIP*::*PDGFRB* fusion transcript, created by an in-frame junction of *MPRIP* exon 20 (17p11.2) and *PDGFRB* exon 12 (5q32). The corresponding genomic breakpoints localized to *MPRIP* at chr17:17079826 and *PDGFRB* at chr5:149505140 (GRCh37/hg19), generating a chimeric fusion protein predicted to retain the intact *PDGFRB* tyrosine kinase domain, consistent with known activating *PDGFRB* rearrangements (Fig. 1C). Complementary DNA-based sequencing using the Myeloid NGS Panel revealed no pathogenic or likely pathogenic variants, indicating the absence of additional cooperating myeloid driver mutations (Table 1S). Together, these molecular findings confirm a pathogenic *MPRIP*::*PDGFRB* gene fusion arising from t(5;17)(q32;p11.2), establishing the oncogenic driver underlying this patient’s eosinophilia-associated myeloid neoplasm.

The patient was initiated on imatinib therapy, given the presence of a *PDGFRB*-rearranged eosinophilia-associated neoplasm. Imatinib was started at a dose of 400 mg daily, with plans for close hematologic and molecular monitoring. Supportive care was provided concurrently, including allopurinol for tumor-lysis prophylaxis, doxycycline for management of his folliculitis skin eruption, nystatin for treatment of oropharyngeal candidiasis, and acyclovir for viral prophylaxis. The patient tolerated therapy without immediate complications, and follow-up assessments were arranged to evaluate hematologic response, molecular clearance of the fusion transcript, and improvement in systemic manifestations. Within one month of therapy, the peripheral blood counts normalized and the rash disappeared. The patient will undergo repeat imaging and a bone marrow biopsy at the 3-month time point from start of therapy and dose reduction of imatinib if remission is proven.

## Discussion

Rearrangements involving *PDGFRB* represent a rare yet clinically important subset of eosinophilia-associated myeloid and myeloid/lymphoid neoplasms. These fusions result in constitutive activation of the *PDGFRB* tyrosine kinase domain through juxtaposition with a wide range of partner genes that typically provide oligomerization motifs enabling ligand-independent signaling [24, 25]. A total of 45 distinct *PDGFRB* fusion partner genes have been reported to date across a wide spectrum of hematologic malignancies (Table 2). These include chronic myelomonocytic leukemia, atypical chronic myeloid leukemia, chronic eosinophilic leukemia, myeloproliferative neoplasms with eosinophilia, myeloid/lymphoid neoplasms with eosinophilia, and Philadelphia chromosome–like B-cell acute lymphoblastic leukemia. Table 2 highlights the remarkable genetic diversity of *PDGFRB* rearrangements, with partner genes spanning multiple chromosomes and disease contexts, reflecting broad biological heterogeneity. Among the reported fusions, *ETV6*::*PDGFRB* and *TRIP11*::*PDGFRB* represent the most frequently described and historically well-characterized fusion types. Despite differences in fusion partners and clinical phenotypes, a unifying feature of *PDGFRB* rearranged neoplasms is their striking sensitivity to TKIs, particularly imatinib, which often induces rapid and durable hematologic and molecular remissions.

Within this broader landscape, *MPRIP*::*PDGFRB* is among the rarest fusion events. Prior to the current report, only three cases had been described worldwide [5, 20, 21]. All reported cases involved a recurrent translocation t(5;17)(q32;p11.2) generating an in-frame fusion of *MPRIP* exon 20 to *PDGFRB* exon 12. Despite differences in age and symptom burden, these patients consistently demonstrated leukocytosis with eosinophilia, hypercellular bone marrow with eosinophilic proliferation, splenomegaly, and complete clinical remission with imatinib therapy. Our comparison of the current patient with these three published cases reveals several shared features and meaningful distinctions. All four affected individuals are males, suggesting a possible male predominance. Ages span from early thirties to eighty years, and all patients exhibited a chronic myeloid neoplasm characterized by leukocytosis and variable degrees of eosinophilia. Anemia was present in three cases, including the current patient. Peripheral blood eosinophilia ranged from moderate in the current patient to marked in previously reported cases. Bone marrow was hypercellular with fewer than 5% blasts across all cases, though the severity of marrow eosinophilia and fibrosis varied (Table 1).

The current patient is distinguished by multisystem involvement, including a pustular folliculitis-like rash, chronic loose stools, tree-in-bud pulmonary nodularity, hepatosplenomegaly, and mucocutaneous candidiasis, whereas previously published cases reported fewer extramedullary manifestations. All cases demonstrated a clonal *PDGFRB* rearrangement with similar proportions of abnormal cells, including 79% in the current patient, 73% in the case described by Beel et al., and 70% in the case described by Naumann et al. All patients received imatinib therapy, and each of the previously published cases achieved complete remission within six to twelve months, and the current patient achieved rapid hematologic and clinical remission within one month, with further evaluation planned at three months to guide possible imatinib dose reduction (Table 1).

In summary, this case report represents the first documented *MPRIP*::*PDGFRB* fusion positive MLN-eo patient in the United States and expands the known clinical spectrum associated with this exceptionally rare fusion. By comparing our findings with the three previously reported cases and reviewing all known *PDGFRB* fusion partners, this report underscores the importance of comprehensive cytogenetic and molecular testing in patients with unexplained eosinophilia. Identification of a *PDGFRB* rearrangement carries critical therapeutic implications due to the remarkable efficacy of tyrosine kinase inhibition and continues to refine our understanding of eosinophilia-associated hematologic neoplasms. 

## Supplementary information

Below is the link to the electronic supplementary material.

Supplementary Material 1

## Data Availability

All relevant cytogenetic, molecular, and clinical data generated for this case have been incorporated into the manuscript. Additional supporting information and raw data underlying the findings of this report are available from the corresponding author upon reasonable request.
