# Supplementary material for: MPRIP::PDGFRB fusion identified in a male patient with a myeloid/lymphoid neoplasm with eosinophilia
Source: Ann Hematol. 2026 Apr 27;105(5):268. doi: 10.1007/s00277-026-07035-8 (PMC13121181; doi:10.1007/s00277-026-07035-8)
Supplement: Supplementary file 1 — Supplementary Material 1 [file 277_2026_7035_MOESM1_ESM.docx]

| **Tests** | **Results** |
| --- | --- |
| **Peripheral blood differential WBC count** | Total WBC count 4.94 × 10¹⁰/L with 75% (3.705 × 10¹⁰/L) neutrophils, 13% (6.422 × 10⁹/L) eosinophils, 1% (4.94 × 10⁸/L) basophils, 1% (4.94 × 10⁸/L) lymphocytes, 4% (1.976 × 10⁹/L) monocytes, and 6% (2.964 × 10⁹/L) myelocytes. The hemoglobin is 105 g/L, and the platelet count 1.095 × 10¹¹/L |
| **Bone Marrow Morphology** | Markedly hypercellular marrow (>95%) with increased eosinophils, dysmegakaryopoiesis, mild reticulin fibrosis (grade 1/3), and <5% blasts with no circulating blasts. |
| **Eosinophilia FISH Panel** | *PDGFRB* rearrangement (79% of cells); *PDGFRA*, *FGFR1*, *JAK2*, and *ETV6*: Negative. |
| **Chromosome Analysis** | 46,XY,t(5;17)(q32;p11.2)[18]/46,XY[2] |
| **NGS Myeloid RNA Fusion Panel** | *MPRIP*::*PDGFRB* fusion; Breakpoints: *MPRIP* (NM_015134.3): chr17, exon 20, position 17079826; *PDGFRB* (NM_002609.3): chr5, exon 12, position 149505140. |
| **NGS Myeloid DNA Panel** | No pathogenic variants detected |

**Table S1. Summary of Diagnostic Laboratory Findings in the Current Patient**
